# Correlation between loneliness, personality traits, and treatment outcomes in patients with methamphetamine use disorder

**DOI:** 10.1038/s41598-022-11901-6

**Published:** 2022-05-23

**Authors:** Tsung-Yu Tsai, Tzu-Yun Wang, Huai-Hsuan Tseng, Kao Chin Chen, Ching-Ju Chiu, Po See Chen, Yen Kuang Yang

**Affiliations:** 1 Department of Psychiatry, Tainan Hospital, Ministry of Health and Welfare, Tainan, Taiwan; 2 Department of Psychiatry, National Cheng Kung University Hospital, College of Medicine, National Cheng Kung University, 138 Sheng-Li Road, Tainan, 70428 Taiwan; 3 Institute of Behavioral Medicine, College of Medicine, National Cheng Kung University, Tainan, Taiwan; 4 Institute of Gerontology, College of Medicine, National Cheng Kung University, Tainan, Taiwan

**Keywords:** Outcomes research, Risk factors, Addiction

## Abstract

The aim of this study was to investigate whether loneliness and personality traits correlate with the treatment outcome of methamphetamine use disorder. In this 1-year longitudinal study, a total 106 participants (98 males, 8 females), with a mean age 36.3 ± 9.6 years were enrolled. We measured UCLA Loneliness Scale and Tridimensional Personality Questionnaire at baseline, while craving level at baseline, week 12, 24, 36, and 48. Urinary methamphetamine tests were given 17 times. For the evaluation of the data, multiple linear regression and generalized linear mixed models were used. The baseline results showed lower levels of the harm avoidance trait and higher levels of loneliness were significantly associated with higher craving levels (p=0.04 and 0.04). Moreover, loneliness was not only positively associated with craving levels (B=0.05, p<0.01) but with urinary methamphetamine positive results (B= 0.08, p=0.03) during one-year treatment. The findings suggested that loneliness was associated with poor methamphetamine treatment outcome (greater craving levels and higher proportion of positive methamphetamine urine tests) and lower harm avoidance traits are associated with higher craving levels.

## Introduction

In the past decades, the use of methamphetamine-type stimulants increased rapidly worldwide. Over the period 2009–2019, the quantities of methamphetamine seized increase almost ten times with a profound increase in East and South East Asia^1^. Those who abused methamphetamine-type stimulants commonly started in their late teenage years or their early twenties. Any application of methamphetamines increases the risk of cardiovascular injury^2^, neurological damage^3^, psychosis, violence, suicidality, depression, and dementia^4,5^. Those who had methamphetamine abuse were more likely to have domestic violence toward their intimate partners^6^, intentional self-inflicted injury or internal assaults^7^, and increased annual hospital costs^8^. Problematic methamphetamine use is less well defined, but methamphetamine use disorder is defined as “a pattern of amphetamine-type substance use leading to clinical significant impairment or distress when at least two of 11 criteria (Diagnostic and Statistical Manual of Mental Disorder, Fifth Edition, DSM-5) within a 12-month period” including a longer period or a larger amount consumption then intended, cravings, tolerance, and withdrawal^9^. Up to date, there is still no effective pharmacotherapy for methamphetamine dependence^10^. Therefore, it is warranted to explore and to identify the complex associations with the psychological and sociodemographic factors for individuals with methamphetamine use disorder.

Several papers across different countries and ethnicities reported personality traits play a vital role in subjective effects and addictive behaviors^11–13^. For example, individuals with lower harm avoidance traits were associated with greater positive activation effects of amphetamine but such association were not observed in those who have higher trait of impulsivity^14^. Individuals who were high in neuroticism and low in conscientiousness were more like to consume drugs^15^. Furthermore, higher levels of antisocial traits and aggressive traits in those who had substance use disorder were significant predictors of higher drop out from treatment^16^. That using Cloninger’s Temperament Scales is an effective tool to explore the correlations between personality traits and substance-related behaviors^17^. Based on Cloninger’s theory of personality, *Cloninger*’*s Tridimensional Personality Questionnaire* consist of 3 domains: Novelty Seeking (NS), Harm Avoidance (HA), and Reward Dependence (RD). In Cloninger’s personality models, NS is characterized by low tolerance to boredom and a willingness to take risks for the sake of stimulations, HA is characterized by higher tendency to responds intensely to aversive stimuli and to learn to passively avoid punishment, and RD is characterized as a tendency to respond markedly to signals of reward^18^. NS and HA are associated with higher risk of substance abuse^17,19^. For example, those who had stimulant abuse reported higher scores of NS, while those who had opioid and hypnotics abuse reported higher scores of HA^20^. In addition, both human and rodent studies demonstrated that high novelty seeking can predict an earlier beginning stage of drug abuse and a transition to compulsive drug use and relapse^21^. Moreover, high levels of HA and NS are also associated with higher risk for the development of methamphetamine dependence among methamphetamine users^22^. Substance abusers with higher level of NS and HA are associated with higher tendencies to suffer from somatic and emotional distress^19^. Nonetheless, there remains debate over RD as a reliable dimension of temperament^23^ and some studies tried to identify personal risk factors to reduce the severity of dependence and the odds of positive urine methamphetamine test in methamphetamine use disorder, their results remain inconclusive^10,24^.

Loneliness is a subjectively distressing feeling when an individual perceives a lack of quality or quantity in interpersonal relationships^25^. A host of reports indicate that loneliness is associated with mental health symptomatology such as depression and anxiety, and addictive behaviors^26^. The feeling of loneliness is stronger with drug abuse than without. The feeling of loneliness increases the odds of abusing drugs and taking part in high risk behaviors^27^. Lonely individuals were found significantly correlated to compulsive internet use, excessive alcohol use, and problem gambling^28^. Furthermore, loneliness increased odds of using illicit opioids among those who received methadone maintenance treatment^29^. These addictive behaviors and drug abuse may damage one's social networks, leading to loneliness in turn^30,31^. Although social supports may assuage loneliness and lead to healthier behaviors, loneliness oppositely diminished the positive association between social support and better health more profoundly^32^. However, the effects of loneliness on reducing the odds of positive urine methamphetamine tests and craving levels among those who have methamphetamine use disorder remain unclear.

In a recent study, the loneliness effects were longitudinally associated with the development of personality traits from late adolescence to early midlife. Loneliness was positively associated with neuroticism and negatively with extraversion and agreeableness^33^. Furthermore, higher levels of loneliness predicted less perceived self-control and a tendency to avoid risks^34^. Loneliness positively correlated with anxiety and negatively with social risk-taking so that loneliness may positively correlate with HA^35^. The rank-order pattern for loneliness was as stable as the personality characteristics and which followed an inverted U-shape trajectory across the life span^36^. In one Korea study, dependent personality significantly correlated with smartphone addiction and loneliness. In advance, loneliness partially mediated the relationship between dependent personality and smartphone addiction^37^. Both personality traits and loneliness are correlated with substance abuse, whereas there are scarce of studies examining the impacts simultaneously of loneliness and personality traits on the treatment outcome of methamphetamine use order.

In the current study, we investigate the associations between loneliness and personality traits, and treatment outcomes in patients with methamphetamine use disorder. We hypothesize that higher loneliness and greater tendency of NS and HA personality traits are associated with poorer treatment outcome of methamphetamine use disorder. We choose the independent variables as loneliness and personality traits, measuring craving levels, and the results of urinary methamphetamine tests as the treatment outcome variables of methamphetamine use disorder.

## Methods

### Study design

This is a 1-year longitudinal study focusing on the effects of loneliness and personality traits on treatment outcomes in those who had methamphetamine use disorder and received the 1-year treatment program.

### Setting

Patients with methamphetamine use disorder were recruited between Jan. 2019 and Dec. 2020 from the addiction clinics of National Cheng Kung University Hospital (NCKUH). All of them received the 3-stage treatment program including physical and psychiatric managements, psychotherapies, and other psychosocial interventions within 1 year. All procedures followed were in accordance with the ethical standards of the responsible committee on human experimentation (the Institutional Review Board at NCKUH, IRB No: A-ER-106-197) and with the Helsinki Declaration of 1975, as revised in 2000. The study procedures were fully explained to each participant before they signed an informed consent.

### Participants

Each participant was interviewed by a board-certified psychiatrist to confirm an initial diagnosis. The first interview was based on the criteria of the Diagnostic and Statistical Manual of Mental Disorders, fifth edition (DSM-5). The second interview was conducted by a well-trained study member to screen other comorbid psychiatric disorders. It was based on the Chinese version of the Mini International Neuropsychiatric Interview (MINI), established on the concepts of DSM-IV. MINI has been widely used in clinical trials and epidemiological studies with an interrater reliability of 0.75^38^.

The participants were between 18 and 65 years old. The participants who were not capable of signing the informed consent or completing the questionnaire, were excluded. Another reason for exclusion was a serious medical or physical condition requiring immediate hospitalization.

### The three-stage treatment program

The treatment program was built on the “Clinical Treatment Guideline for Schedule II Substance Users” (Taiwan Ministry of Health and Welfare, 2017; http://www.dep.mohw.gov.tw/DOMHAOH/cp-4097-43400-107.html).

During the first treatment stage of 4 weeks, the patients received diagnostic interviews to evaluate their substance use history and comorbidity of physical and psychiatric condition. Their demographic data, social functions, family support, and psychological functions were also evaluated during this period. The participants also received weekly outpatient follow-up and urine methamphetamine tests to monitor treatment response.

During the second treatment stage of 8 weeks, the patients received both a supportive and a 12-session group psychotherapy based on a cognitive behavior therapeutic approach. Psychoeducation and service linkages were also provided during this period. This second stage of the treatment program was only provided if the latest previous urinary methamphetamine results were negative. The patients kept receiving biweekly urine methamphetamine tests at an outpatient clinic.

During the third treatment stage of 36 weeks, the patients received a supportive psychotherapy, a 12-session group psychotherapy, and motivation enhancements to maintain methamphetamine abstinence. The urinary methamphetamine tests were given once a month during this period. Case managers were responsible for the treatment integration and monitored during all 3 stages.

### Measures

#### UCLA loneliness scale and tridimensional personality questionnaire at baseline

##### UCLA loneliness

We assessed the level of loneliness through the UCLA Loneliness Scale (version 3), which is a widely used loneliness measure of high reliability and validity^39^. This test contains 20 items. The Cronbach’s alpha of this sample was 0.65. Participants rated how often they felt the way described in the item using a four-point Likert scale ranging from ‘never’ to ‘often’. Higher loneliness scores indicated greater loneliness.

##### Tridimensional personality questionnaire

We assessed the main personality dimensions through the Tridimensional Personality Questionnaire (TPQ) is a self-report personality inventory to measure three major personality dimensions: Novelty Seeking (NS), Harm Avoidance (HA), and Reward Dependence (RD). We used the Chinese version for evaluating the participants’ personality^40^. The Cronbach’s alpha values were as follows: for NS, Cronbach’s α = 0.72, for HA, Cronbach’s α = 0.89, and for RD, Cronbach’s α = 0.54. In the present study, we only measured NS and HA because the RD dimension was not reliable for Han Chinese in Taiwan^40^.

#### Outcome measurement: Visual Analogue Scale and urine methamphetamine test

We assessed the outcome variable through the Visual Analogue Scale (VAS) and methamphetamine urinalysis. During 1-year treatment program, each participant received 5 sets of Visual Analogue Scale (VAS) and 17 times urine methamphetamine tests.

##### Visual Analogue Scale

The VAS was used to assess the craving level of amphetamine by asking the participants: “How much did you crave/desire/want methamphetamine in the preceding week?”^10^. The participants responded the question with a 100-point Likert scale ranging from 0 (none) to 100 (very much) which was measured at baseline, weeks 12, 24, 36, and at the end of the 1-year treatment program to evaluate the craving levels of using methamphetamine.

##### Urine methamphetamine test

The patient provided their urine sample at each outpatient visit and a total of 17 binary methamphetamine urinalysis results were collected from each participant during the 1-year treatment program (first stage: 4 times once/week; second stage: 4 times once/2 weeks, third stage: 9 times once/4 weeks). Their urine samples were analyzed by using the Amphetamines II (AMPS2)^®^ which is an in vitro diagnostic test for the qualitative and semiquantitative detection of methamphetamines in human urine on the Roche/Hitachi cobas c systems. The cutoff point of amphetamine concentration were 300 ng/ml, 500 ng/ml, and 1000 ng/ml when d-methamphetamine was calibrated.

#### Covariates

Less perceived social support and poorer family relationships were associated with greater loneliness and more severe depression, which had been demonstrated to correlate with poorer recovery/remission of addiction^41,42^. We measured the social support function, family adaptation, partnership, growth, affection, the resolve index (APGAR), and the Hamilton Depression Rating Scale (HDRS) as covariates at baseline.

##### Measurement of support function

The self-reported Measurement of Support Function (MSF) questionnaire had been used to assess social support status. The validity of this assessment in Taiwan was approved by previous studies. In this study, the Cronbach’s alpha of the 20 items was 0.83. This questionnaire consisted of four areas: perceived crisis support, perceived routine support, received crisis support, and received routine support. A higher score indicated more social support^43^.

##### Family adaptation, partnership, growth, affection, and resolve index

The Family Adaptation, Partnership, Growth, Affection, and Resolve (APGAR) index is composed of five domains: Adaptability, Partnership, Growth, Affection, and Resolve^44^. The Chinese version of family APGAR index has been validated in Taiwan^45^. The participants responded to the follow questions: “Are you satisfied with the help that you received from your family?,” “Do you talk with your family about your household problems?,” “Do you feel that your family loves you?,” “Are you satisfied with the time that you and your family share together?,” and “Do you discuss with your family about important decisions that affect the whole family?.” Each domain was answered along a 4-point Likert scale ranging from 1 (low satisfaction) to 4 (high satisfaction), thus the total scores ranged from 4 to 20. Higher score represented better family functions.

##### Hamilton Depression Rating Scale

The 17-item HDRS is for the measurement of the severity of depressive symptoms. This assessment provides comprehensive coverage of depressive symptoms. It has strong psychometric properties, high concurrent and differential validity, and strong reliability. The participants had to rate 11 items for the severity of depressive symptoms along a three- to five-point Likert scale. The total score ranged from 0 to 52. The Cronbach’s alpha of this sample was 0.77^46^. The higher scores of the 17-item HDRS indicated greater severity of depressive symptoms.

#### Baseline demographic and clinical characteristics

We collected the participants’ baseline demographic characteristics including age, sex, educational attainment, and marital status. The age and educational attainment were collected as continuous variables. We also recorded the duration of methamphetamine use by asking the participant: “How many years have you used methamphetamine?”. In addition, according to the evaluation results of MINI, the psychiatric comorbidities were itemized into 5 binary categories: Mood disorder (Yes/No), Anxiety disorder (Yes/No), Psychotic disorder (Yes/No), other substance use disorder (Yes/No), and Anti-social personality disorder (Yes/No). In each category, one point was assigned if the result was yes. Based on the sum of these 5 category result, the psychiatric comorbidity index is ranging from 0 to 5 and higher scores of psychiatric comorbidity index indicated more psychiatric comorbidities.

### Statistical analysis

The analysis for obtaining descriptive statistics and a multiple linear regression was carried out with SAS 9.4 statistical software. Descriptive analysis was performed for all variables at baseline. If the distribution of the variable x was different from normal, we transformed log (x + 1) into normal distribution. Independent samples *t* tests or Chi-squared tests were used to assess associations between the demographic data and the outcome variables.

Because the VAS and urine methamphetamine tests had to be assessed repeatedly, the generalized linear mixed models (GLMMs) method was used for obtaining multiple linear regression analysis. GLMMs in repeated-measures analyses that accommodate randomly missing data^47^. Furthermore, hierarchical regression models were applied to investigate the association between loneliness, personality traits (TPQ-HA and TPQ-NS), and the outcome variables (VAS and urine methamphetamine test). For each outcome variable, three models were estimated at first stage and the endpoint during the 1-year treatment program. In Model 1, we regressed loneliness and personality traits (TPQ-HA and TPQ-NS) on the outcome variable. In Model 2, we then repeated the analyses, adjusting for age and education. In Model 3, we repeated the analyses adjusting for disease duration, psychiatric comorbidities, MSF, HDRS, family APGAR, and the numbers of visits.

 All tests were evaluated at a 0.05 level of statistical significance.

## Results

Table 1 summarized the participant characteristics at baseline and the endpoint. At baseline, there were 106 methamphetamine use patients enrolled (male: 92.4% and mean duration using methamphetamine: 6.5 ± 7.3 years). About fifty percent of the patients had psychiatric comorbidities other than methamphetamine use disorder. The independent variables of baseline loneliness score, TPQ-HA, and TPQ-NS score, and the outcome variables of baseline VAS score and the proportion of positive methamphetamine urine tests were listed in Table 1.

**Table 1:** Participant characteristics at baseline and endpoint.

Variable	Baseline	After 52 weeks	Paired *t* or χ^2^	p
Cases (n)	106	41		
Age (years, mean ± SD)	36.3 ± 9.6	39.7 ± 10.2	–	–
Sex (male/female)	98/8	39/2	–	–
Education (years, mean ± SD)	12.6 ± 2.9	12.7 ± 2.8		
Disease duration (years, mean ± SD)	6.5 ± 7.3	7.7 ± 7.9	–	–
**Psychiatric comorbidities (n/%)**			–	–
Yes	60 (56.6)	–	–	–
No	46 (43.4)	–	–	–
**Mood disorders (n/%)**			–	–
Yes	37 (34.9)	–	–	–
No	69 (65.1)	–	–	–
**Anxiety disorder (n/%)**			–	–
Yes	18 (17.0)	–	–	–
No	88 (83)	–	–	–
**Psychotic disorder (n/%)**				
Yes	8 (7.5)	–		
No	98 (92.5)	–		
**Other SUD (n/%)**			–	–
Yes	41 (38.7)	–	–	–
No	65 (61.3)	–	–	–
**ASPD (n/%)**			–	–
Yes	12 (11.3)	–	–	–
No	94 (88.7)	–	–	–
**Urine methamphetamine (n/%)**			114.6	0.007
Positive	25 (23.6)	2 (5.0)	–	–
Negative	81 (76.4)	26 (63)	–	–
Loneliness (mean ± SD)	43.3 ± 10.3	–	–	–
TPQ-HA (mean ± SD)	14.4 ± 5.4	–	–	–
TPQ-NS (mean ± SD)	16.0 ± 4.2	–	–	–
MSF (mean ± SD)	102.1 ± 18.2	–	–	–
HDRS (mean ± SD)	5.4 ± 4.2	–	–	–
APGAR (mean ± SD)	12.5 ± 4.8	–	–	–
VAS (mean ± SD)^#^	13.2 ± 21.0	9.8 ± 18.1	2.27	0.03

*SUD* substance use disorder, *ASPD* anti-social personality disorder, *TPQ* tridimensional personality questionnaire, *HA* harm avoidance, *NS* novelty seeking, *MSF* measure of support function, *HDRS* Hamilton depression rating scale, *APGAR* family adaptation, partnership, growth, affection, and resolve index, *VAS* visual analog scale (# reported original scores).

After 52 weeks of methamphetamine treatment, 41 out of 106 patients (male: 95.1%; and mean duration using methamphetamine: 7.7 ± 7.9 years) completed a total of 5 times of the VAS scores, but only 28 of them completed a total of 17 times of urine methamphetamine test. The latest VAS score and the proportion of positive urinary methamphetamine tests were lower than at baseline.

Table 2 displays the initial associations between independent variables and dependent variables, we collected the first stage of treatment outcome parameters for hierarchical regression analysis. Model 1 showed a positive association between loneliness and craving level (B = 0.05, p = 0.02) but a negative association between TPQ-HA and craving level (B = − 0.10, p = 0.01). In Model 2, adjusting for age and education, the positive association between loneliness and craving level (B = 0.05, p = 0.03) and the negative association between TPQ-HA and craving level (B = − 0.10, p = 0.01) remained consistent. In Model 3, the positive association between loneliness and craving level (B = 0.05, p = 0.04) and the negative association between TPQ-HA and craving level (B = − 0.09, p = 0.04) are consistent after adjusting for disease duration, psychiatric comorbidities, MSF, HDRS, family APGAR, and the numbers of visits remained robust. However, TPQ-NS was not significantly associated with craving level. We also examined the association between the independent variables and 4 times urine methamphetamine tests in total by GLMMs analysis. We found no significant association between the baseline of the independent variables and the first stage of urine methamphetamine tests.

**Table 2:** Association between loneliness, personality traits, and craving level for methamphetamine, and the urinary methamphetamine test results at first stage in methamphetamine-use patients.

	Craving level^a^			Urine methamphetamine test result		
	B	*t* (95% CI)	p	B	*t* (95% CI)	p
**Model 1: Unconditional**						
Loneliness	**0.05**	**2.38 (0.01 to 0.09)**	0.02*	− 0.02	− 0.62 (− 0.08 to 0.04)	0.53
TPQ-HA	− **0.10**	− **2.50 (**− **0.17 to **− **0.02)**	0.01*	0.05	0.86 (− 0.07 to 0.17)	0.39
TPQ-NS	0.07	1.80 (− 0.01 to 0.14)	0.07	− 0.01	− 0.22 (− 0.13 to 0.10)	0.83
**Model 2: Age and education adjusted**						
Loneliness	**0.05**	**2.26 (0.01 to 0.09)**	0.03*	− 0.03	− 0.80 (− 0.09 to 0.04)	0.42
TPQ-HA	− **0.10**	− **2.49 (**− **0.17 to **− **0.02)**	0.01*	0.06	0.92 (− 0.06 to 0.18)	0.36
TPQ-NS	0.06	1.58 (− 0.02 to 0.14)	0.12	− 0.01	− 0.23 (− 0.14 to 0.11)	0.82
**Model 3: Full adjusted**^**b**^						
Loneliness	**0.05**	**2.05 (0.002 to 0.10)**	0.04*	− 0.06	− 1.42 (− 0.15 to 0.02)	0.16
TPQ-HA	− **0.09**	− **2.07 (**− **0.17 to **− **0.003)**	0.04*	0.04	0.60 (− 0.11 to 0.19)	0.60
TPQ-NS	0.02	0.04 (− 0.06 to 0.11)	0.57	0.03	0.35 (− 0.12 to 0.04)	0.73

*p < 0.05, **p < 0.01.

*TPQ* tridimensional personality questionnaire, *HA* harm avoidance, *NS* novelty seeking.

^a^Transformed VAS by log (x + 1).

^b^Model 3: adjusting for age, education, disease duration (years), psychiatric comorbidities, measure of support function (MSF), Hamilton depression rating scale (HDRS), family adaptation, partnership, growth, affection, and resolve index (family APGAR), and the numbers of visits.

In advance, as shown in Table 3, we examined the associations between the baseline independent variables and the repetitive outcome variables, 5 sets of VAS scores and 17 urine methamphetamine tests, during the 1-year treatment program by GLMMs analysis. Model 1 on craving levels showed that loneliness was positively associated with greater levels of craving (B = 0.05, p = 0.003) but TPQ-HA was negatively associated with greater level of craving level (B = − 0.07, p = 0.02). Model 2 further added age and education, and the positive association between loneliness and craving level (B = 0.05, p = 0.003) and the negative association between TPQ-HA and craving level (B = − 0.07, p = 0.02) were consistent. Model 3 further adjusted disease duration, psychiatric comorbidities, MSF, HDRS, family APGAR, and the numbers of visits, the positive association between loneliness and craving level (B = 0.05, p = 0.03) and the negative association between TPQ-HA and craving level (B = − 0.07, p = 0.03) remained robust. However, in Model 3, the association between TPQ-NS and craving level was not significant (B = 0.05, p = 0.17).

**Table 3:** Association between baseline loneliness, personality traits, craving level for methamphetamine, and urinary methamphetamine test results during the 1-year treatment program in methamphetamine-use patients.

	Craving level^a^			Urine methamphetamine test result		
	B	*t* (95% CI)	p	B	*t *(95% CI)	p
**Model 1: Unconditional**						
Loneliness	**0.05**	**3.06 (0.02 to 0.08)**	0.003**	0.04	1.63 (− 0.01 to 0.10)	0.10
TPQ-HA	− **0.07**	− **2.39 (**− **0.13 to **− **0.01)**	0.02*	− 0.06	− 1.22 (− 0.16 to 0.04)	0.22
TPQ-NS	**0.08**	**2.61 (0.02 to 0.14)**	0.01*	0.05	1.00 (− 0.05 to 0.14)	0.32
**Model 2: Age and education adjusted**						
Loneliness	**0.05**	**2.99 (0.02 to 0.08)**	0.003**	0.05	1.76 (− 0.01 to 0.10)	0.08
TPQ-HA	− **0.07**	− **2.42 (**− **0.13 to **− **0.01)**	0.02*	− 0.07	− 1.33 (− 0.17 to 0.03)	0.18
TPQ-NS	**0.07**	**2.26 (0.01 to 0.14)**	0.02*	0.04	0.82 (− 0.06 to 0.15)	0.41
**Model 3: Full adjusted**^**b**^						
Loneliness	**0.05**	**2.26 (0.01 to 0.08)**	0.03*	**0.08**	**2.04 (0.003 to 0.15)**	0.04*
TPQ-HA	− **0.07**	− **2.18 (**− **0.14 to **− **0.01)**	0.03*	− 0.06	− 0.96 (− 0.18 to 0.06)	0.34
TPQ-NS	0.05	1.39 (− 0.02 to 0.11)	0.17	0.01	0.15 (− 0.11 to 0.13)	0.88

*p < 0.05, **p < 0.01.

*TPQ* tridimensional personality questionnaire, *HA* harm avoidance, *NS* novelty seeking.

^a^Transformed VAS by log (x + 1).

^b^Model 3: adjusting for age, education, disease duration (years), psychiatric comorbidities, measure of support function (MSF), Hamilton depression rating scale (HDRS), family adaptation, partnership, growth, affection, and resolve index (family APGAR), and the numbers of visits.

Although, during 1-year program, Model 1 and Model 2 showed loneliness and personality traits insignificantly associated with positive urinary methamphetamine results, loneliness positively associated positive urinary methamphetamine results after adjusting disease duration, psychiatric comorbidities, MSF, HDRS, family APGAR, and the numbers of visits in Model 3 (B = 0.08, p = 0.04) (Table 3).

## Discussion

In the present study, we hypothesized that higher loneliness and greater tendency of NS and HA personality traits were associated with greater craving levels of methamphetamine and higher proportion of positive urine methamphetamine results in those who attended the 1-year treatment program for methamphetamine use disorder. In accord with the hypothesis, our findings suggested that greater loneliness was associated with greater craving levels of methamphetamine and higher proportion of positive urine methamphetamine results. Not only at the first stage but at the end of treatment program remained the consistent findings between loneliness and the treatment outcomes of craving and positive urine methamphetamine results. However, inconsistent with the hypothesis, we found a negative association between HA personality trait and craving level of methamphetamine and a nonsignificant association between NS personality trait and craving level of methamphetamine. Neither HA personality trait nor NS personality were insignificantly associated with an increasing numbers of positive urine methamphetamine results.

Loneliness impacts social experiences and emotional states. It weakens the immune system and increases stress-related responses. When one feels lonely, a greater perceived loneliness is associated with increased ventral stratum and midbrain activity, contributing to “social craving” in a similar way as fasting causes hunger^50,51^. In parallel, it was previously shown that a negative emotional state leads to a change in the extended amygdala and increases perpetuation of drug-taking behavior to remunerate the negative feeling^52^. Those who experience loneliness have higher likelihood of depression and anxiety and are more likely to engage in impulsive behaviors such as unplanned spending and over-eating^53,54^. Both craving and stress relief trigger lonely individuals to search for the drug and impaired prefrontal top-down self-regulation diminishes their awareness of addiction^52^.

Our results suggest that lonely methamphetamine users have poor treatment response and higher tendency to use methamphetamine even they are under deferred prosecution status with close monitoring. We find that higher levels of loneliness are associated with higher craving levels of methamphetamine. Such association are observed both at baseline and during the 1-year methamphetamine treatment program in those participants with methamphetamine use disorder. In addition to higher craving levels, higher levels of loneliness are also associated with higher probability of using methamphetamine.

Compare with NS traits, we find lower HA traits were significantly associated with higher craving level of methamphetamine among those who received methamphetamine treatment program. In the previous study, NS and HA personality are correlated to gray’s biopsychological theory, the behavioral activation system (BAS) and behavioral inhibition system (BIS) respectively^55^. Once individuals know that a reward is likely to follow, those who had higher tendency of NS traits arise their behavioral action system to carry out some behaviors to attain reward when they were exposed to the environment cues of the drug. They also had higher level of craving and long-term vulnerability of relapse of psychostimulant drug abuse^56^. However, when individuals developed with psychostimulant drug dependence, avoiding aversive feeling passively instead of achieving rewards actively may be more significantly lead them to use psychostimulant drug^57,58^. Although people with higher levels of HA are associated with lower social adaption and higher risk for affective disorders^59^, people with lower levels of HA have difficulties in avoiding aversive outcomes when looking for immediate monetary rewards^60^.

There were several limitations in the present study. (1) The sample size was medium, and the attrition rate was high. Only 39% of the participants completed the 1-year longitudinal evaluation and during the 1-year treatment program we collected: 68.1% of craving level evaluations (106 participants, 5 test occasions, 361 out of 530 samples); 66.7% of urinary methamphetamine test results (106 participants, 17 test occasions, 1202 out of 1802 samples). (2) Over 95% of the participants in this study were convicted of methamphetamine abuse and underwent the deferred prosecution program, which simultaneously request them to attend the 1-year treatment-based program. Although we had explained to the participants that the assessments in the current study were not related with their legal issue, some participants may under-report their severity of craving. (3) Although the evidence of personality change following substance use remains inconsistent^15^, it is possible that methamphetamine use may change the personality and the personality changes may persisted over the course of the study as participants were treated^61^. These results need to be interpreted cautiously to determining the long-term outcomes. (4) Because of limited completion of measurement during the 1-year program, we only used the first stage of measurements in the mediation analysis about the role of loneliness between personality and craving level. The mediation effect of loneliness on the association between personality and craving level needs to be verified again in future studies.

## Conclusion

We found loneliness is associated with poorer methamphetamine treatment outcomes. Higher loneliness levels were associated with higher craving levels and higher tendency of methamphetamine use. In addition, lower HA personality is associated with higher craving levels of methamphetamine. Additional studies on regulating loneliness to improve treatment outcomes in methamphetamine use patients may be warranted.

## References

[1] United Nations Office on Drug and Crime. *World Drug Report 2021*. https://www.unodc.org/unodc/en/data-and-analysis/wdr2021.html (2021).

[2] Bazmi E (2017). Cardiovascular complications of acute amphetamine abuse: Cross-sectional study. Sultan Qaboos Univ. Med. J..

[3] Silva AP (2010). Brain injury associated with widely abused amphetamines: Neuroinflammation, neurogenesis and blood–brain barrier. Curr. Drug Abuse Rev..

[4] McKetin R (2019). Mental health outcomes associated with the use of amphetamines: A systematic review and meta-analysis. EClinicalMedicine.

[5] Tzeng N-S (2020). Association between amphetamine-related disorders and dementia—A nationwide cohort study in Taiwan. Ann. Clin. Transl. Neurol..

[6] Morgan A, Gannoni A (2020). Methamphetamine dependence and domestic violence among police detainees. Trends Issues Crime Crim. Justice.

[7] Tominaga GT (2004). Toll of methamphetamine on the trauma system. Arch. Surg..

[8] Winkelman TNA (2018). Evaluation of amphetamine-related hospitalizations and associated clinical outcomes and costs in the United States. JAMA Netw. Open.

[9] Hamel C (2020). Psychosocial and pharmacologic interventions for methamphetamine addiction: Protocol for a scoping review of the literature. Syst. Rev..

[10] Siefried KJ (2020). Pharmacological treatment of methamphetamine/amphetamine dependence: A systematic review. CNS Drugs.

[11] Shahini N (2021). Temperament and character traits in substance use disorder in Iran: A case control study. BMC Psychol..

[12] Seyed Hashemi SG (2019). Personality traits and substance use disorders: Comparative study with drug user and non-drug user population. Pers. Individ. Differ..

[13] Hojjat SK (2015). Personality traits and identity styles in methamphetamine-dependent women: A comparative study. Global J. Health Sci..

[14] White TL, Lott DC, de Wit H (2006). Personality and the subjective effects of acute amphetamine in healthy volunteers. Neuropsychopharmacology.

[15] Kroencke L (2020). How does substance use affect personality development? Disentangling between- and within-person effects. Soc. Psychol. Pers. Sci..

[16] Papamalis FE (2020). Examining the relationship of personality functioning and treatment completion in substance misuse treatment. Subst. Abuse Res. Treat..

[17] Hartman C (2013). Using Cloninger's temperament scales to predict substance-related behaviors in adolescents: A prospective longitudinal study. Am. J. Addict..

[18] Cloninger CR, Svrakic DM, Przybeck TR (1993). A psychobiological model of temperament and character. Arch. Gen. Psychiatry.

[19] Ma CH (2020). Specific personality traits and associated psychosocial distresses among individuals with heroin or methamphetamine use disorder in Taiwan. J. Formos Med. Assoc..

[20] Hurtado G (2016). Personality dimensions and drug of choice: A descriptive study using Cloninger's temperament and character inventory revised. Eur. Psychiatry.

[21] Wingo T (2016). Novelty seeking and drug addiction in humans and animals: From behavior to molecules. J. Neuroimmune Pharmacol..

[22] Tzeng N-S (2015). The dopamine transporter gene may not contribute to susceptibility and the specific personality traits of amphetamine dependence. Drug Alcohol Depend..

[23] Gillespie NA (2003). The genetic and environmental relationship between Cloninger's dimensions of temperament and character. Pers. Individ. Differ..

[24] Khoramizadeh M (2019). Treatment of amphetamine abuse/use disorder: A systematic review of a recent health concern. Daru.

[25] Weiss RS (1987). Reflections on the present state of loneliness research. J. Soc. Behav. Pers..

[26] Hawkley LC, Cacioppo JT (2010). Loneliness matters: A theoretical and empirical review of consequences and mechanisms. Ann. Behav. Med..

[27] Hosseinbor M (2014). Emotional and social loneliness in individuals with and without substance dependence disorder. Int. J. High Risk Behav. Addict..

[28] Savolainen I (2020). The role of perceived loneliness in youth addictive behaviors: Cross-national survey study. J. Med. Internet Res..

[29] Polenick CA (2019). Loneliness and illicit opioid use among methadone maintenance treatment patients. Subst. Use Misuse.

[30] Christie NC (2021). The role of social isolation in opioid addiction. Soc. Cogn. Affect. Neurosci..

[31] Wootton RE (2021). Bidirectional effects between loneliness, smoking and alcohol use: Evidence from a Mendelian randomization study. Addiction.

[32] Segrin C, Passalacqua SA (2010). Functions of loneliness, social support, health behaviors, and stress in association with poor health. Health Commun..

[33] Mund M, Neyer FJ (2018). Loneliness effects on personality. Int. J. Behav. Dev..

[34] Zhu Y, Wang C (2017). The lonelier, the more conservative? A research about loneliness and risky decision-making. Psychology.

[35] MacDonald KB, Schermer JA (2021). Loneliness unlocked: Associations with smartphone use and personality. Acta Physiol. (Oxf).

[36] Mund M (2020). The stability and change of loneliness across the life span: A meta-analysis of longitudinal studies. Pers. Soc. Psychol. Rev..

[37] Ok J (2016). The mediating effects of loneliness on the relationship between smartphone addiction and dependent personality trait in adults. Korea J. Health Promot..

[38] Sheehan DV (1998). The Mini-International Neuropsychiatric Interview (M.I.N.I.): The development and validation of a structured diagnostic psychiatric interview for DSM-IV and ICD-10. J. Clin. Psychiatry.

[39] Russell DW (1996). UCLA Loneliness Scale (Version 3): Reliability, validity, and factor structure. J. Pers. Assess..

[40] Chen WJ (2002). Cloninger's Tridimensional Personality Questionnaire: Psychometric properties and construct validity in Taiwanese adults. Compr. Psychiatry.

[41] Wang J (2018). Associations between loneliness and perceived social support and outcomes of mental health problems: A systematic review. BMC Psychiatry.

[42] Kelly SM (2010). The relationship of social support to treatment entry and engagement: The Community Assessment Inventory. Subst. Abuse.

[43] Lin N, Ye X, Ensel WM (1999). Social support and depressed mood: A structural analysis. J. Health Soc. Behav..

[44] Smilkstein G (1978). The family APGAR: A proposal for a family function test and its use by physicians. J. Fam. Pract..

[45] Chau, T.T., S.F. Hsieh, and H.W. Liu, [The use of extended family APGAR index in OPD]. Gaoxiong Yi Xue Ke Xue Za Zhi, **7**(2):75–80 (1991). 2005675

[46] Zheng YP (1988). Validity and reliability of the Chinese Hamilton Depression Rating Scale. Br. J. Psychiatry.

[47] Ibrahim JG, Molenberghs G (2009). Missing data methods in longitudinal studies: A review. Test (Madrid, Spain).

[48] Baron RM, Kenny DA (1986). The moderator-mediator variable distinction in social psychological research: Conceptual, strategic, and statistical considerations. J. Pers. Soc. Psychol..

[49] Shrout PE, Bolger N (2002). Mediation in experimental and nonexperimental studies: New procedures and recommendations. Psychol. Methods.

[50] Tomova L (2020). Acute social isolation evokes midbrain craving responses similar to hunger. Nat. Neurosci..

[51] Inagaki TK (2016). Yearning for connection? Loneliness is associated with increased ventral striatum activity to close others. Soc. Cogn. Affect. Neurosci..

[52] Volkow ND, Michaelides M, Baler R (2019). The neuroscience of drug reward and addiction. Physiol. Rev..

[53] Peng, L., Xia, X. & Su, X. The effect of consumer’s loneliness on impulse buying in the internet era: A model based on para-social interaction. In *2020 2nd International Conference on Economic Management and Model Engineering *(*ICEMME*) (2020).

[54] Tatsi E (2019). Emotion dysregulation and loneliness as predictors of food addiction. J. Health Soc. Sci..

[55] Mardaga S, Hansenne M (2007). Relationships between Cloninger's biosocial model of personality and the behavioral inhibition/approach systems (BIS/BAS). Pers. Individ. Differ..

[56] Arenas MC (2016). Influence of the novelty-seeking endophenotype on the rewarding effects of psychostimulant drugs in animal models. Curr. Neuropharmacol..

[57] Franques P, Auriacombe M, Tignol J (2000). Addiction and personality. Encephale.

[58] Koob GF, Volkow ND (2010). Neurocircuitry of addiction. Neuropsychopharmacology.

[59] Tse WS, Bond AJ (2005). The Application of the Temperament and Character Inventory (TCI) in predicting general social adaptation and specific social behaviors in a dyadic interaction. J. Appl. Soc. Psychol..

[60] Howe LK, Finn PR (2020). The influence of harm avoidance and impulsivity on delay discounting rates. Pers. Individ. Differ..

[61] Wada K, Fukui S (1990). Relationship between years of methamphetamine use and symptoms of methamphetamine psychosis. Arukoru Kenkyuto Yakubutsu Ison.

